# Supplementary material for: MS CETSA deep functional proteomics uncovers DNA repair programs leading to gemcitabine resistance
Source: Nat Commun. 2025 May 7;16:4234. doi: 10.1038/s41467-025-59505-8 (PMC12059070; doi:10.1038/s41467-025-59505-8)
Supplement: Supplementary file 1 — Supplementary Information [file 41467_2025_59505_MOESM1_ESM.pdf]

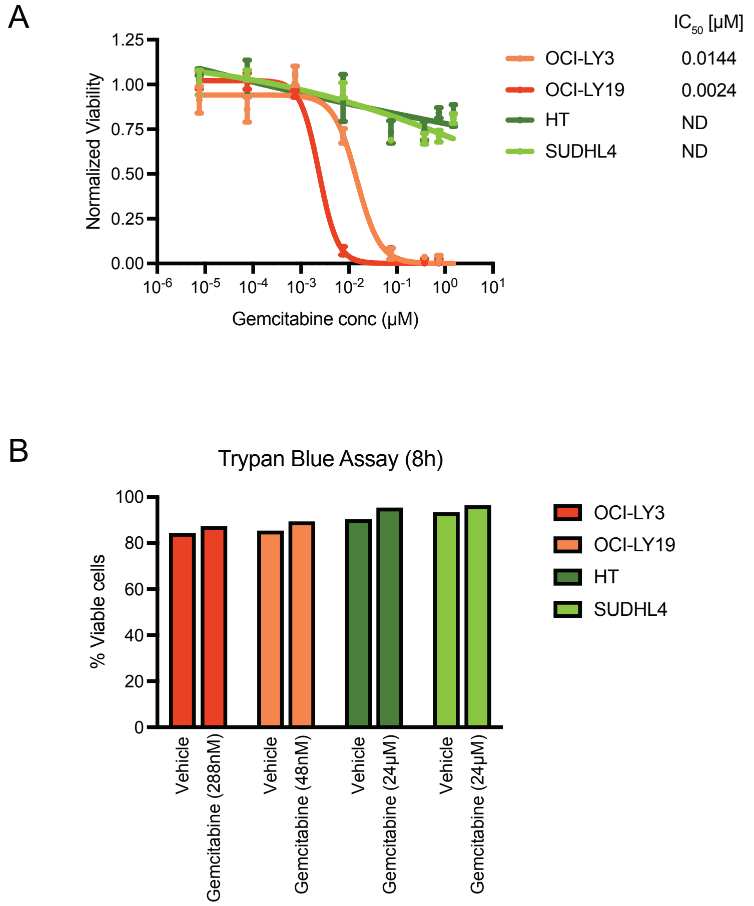

Supplementary Figure 1: Cell viability after gemcitabine treatment

(A) MTT viability assay and IC_{50} values of OCI-LY3 (orange), OCI-LY19 (red), HT (dark green) and SUDHL4 (light green) cells after 48h treatment with increasing concentrations of gemcitabine. Data are presented as mean relative viability compared to the reference ±SEM from biological replicates (n=3). Source data are provided as a Source Data file. (B) Trypan blue assay of OCI-LY3 (orange), OCI-LY19 (red), HT (dark green) and SUDHL4 (light green) cells after 8h treatment with indicated gemcitabine concentrations. Source data are provided as a Source Data file.

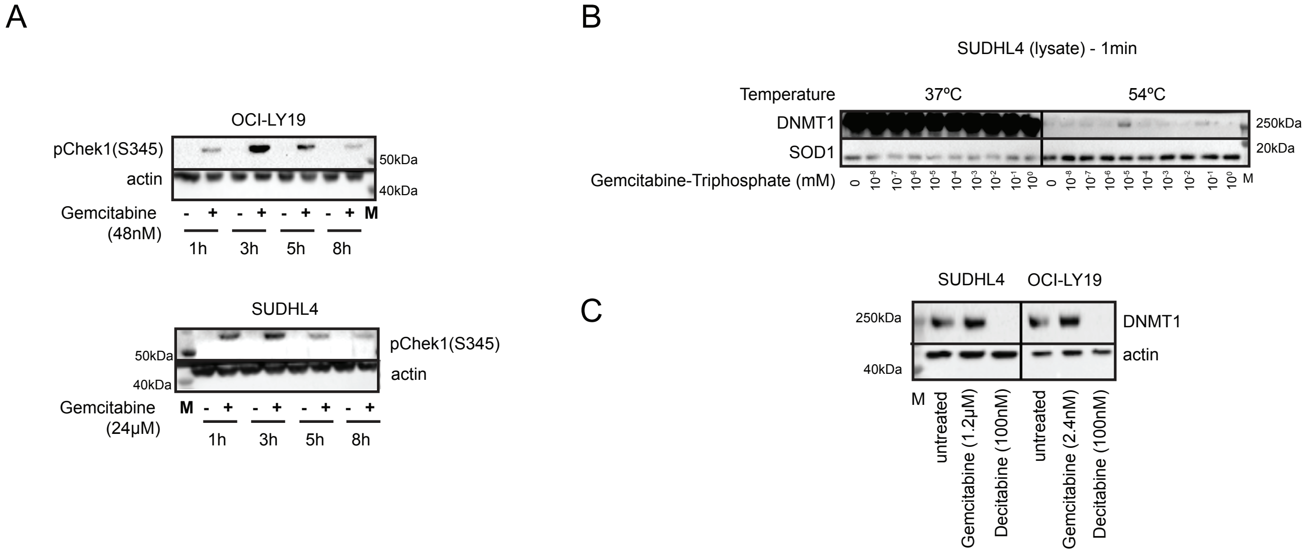

Supplementary Figure 2: Early responses to gemcitabine in resistant and sensitive cells
(A) Western blot detection of phospho-Chk1 (S345) in OCI-LY19 (top) and SUDHL4 (bottom) after treatment with indicated gemcitabine concentrations for 1h, 3h, 5h or 8h. Actin was used as loading control. Source data are provided as a Source Data file. (B) Western blot detection of DNMT1 levels in soluble fraction in SUDHL4 lysates treated with increasing gemcitabine concentrations (0-1mM) for 1min and upon CETSA heat challenge at 37˚C (control) and 54˚C. Source data are provided as a Source Data file.(C) Western blot detection of DNMT1 levels in SUDHL4 (left) and OCI-LY19 (right) cells after 24h treatment with vehicle, gemcitabine or decitabine. Actin was used as loading control. Source data are provided as a Source Data file.

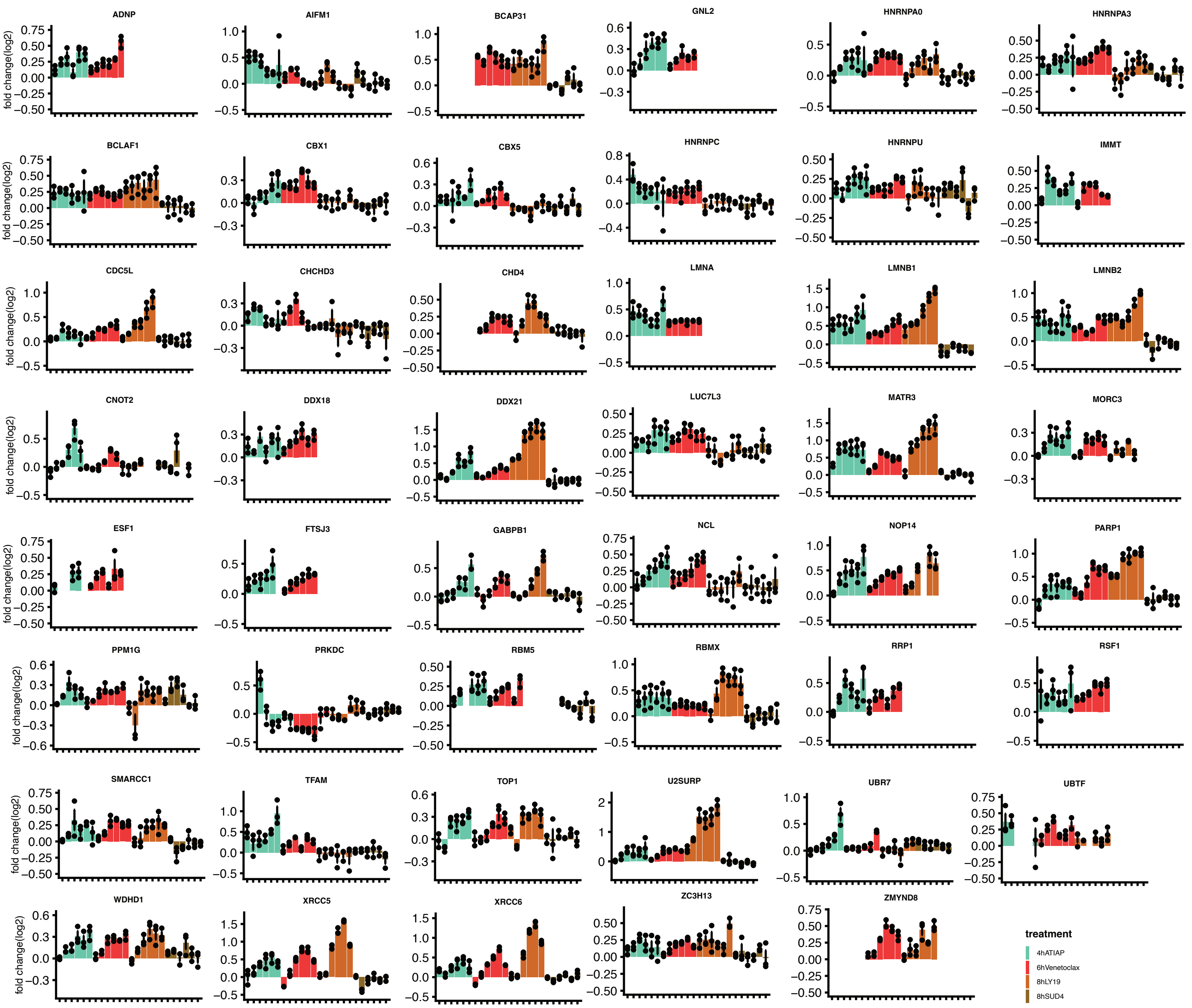

Supplementary Figure 3: Comparison with Core CETSA Apoptosis Ensemble
IMPRINTS profiles of CCAE proteins in MOLM-16 cells treated for 4h with 100nM AT-IAP (Ramos et al), Kasumi-1 cells treated for 6h with 100nM venetoclax (Ramos et al), sensitive OCI-LY19 cells treated for 8h with 48nM gemcitabine and resistant SUDHL4 cells treated for 8h with 24μM gemcitabine. Data are presented as mean log2 fold change compared to the reference ±SEM from biological replicates (n=3). Source data are provided as a Source Data file.

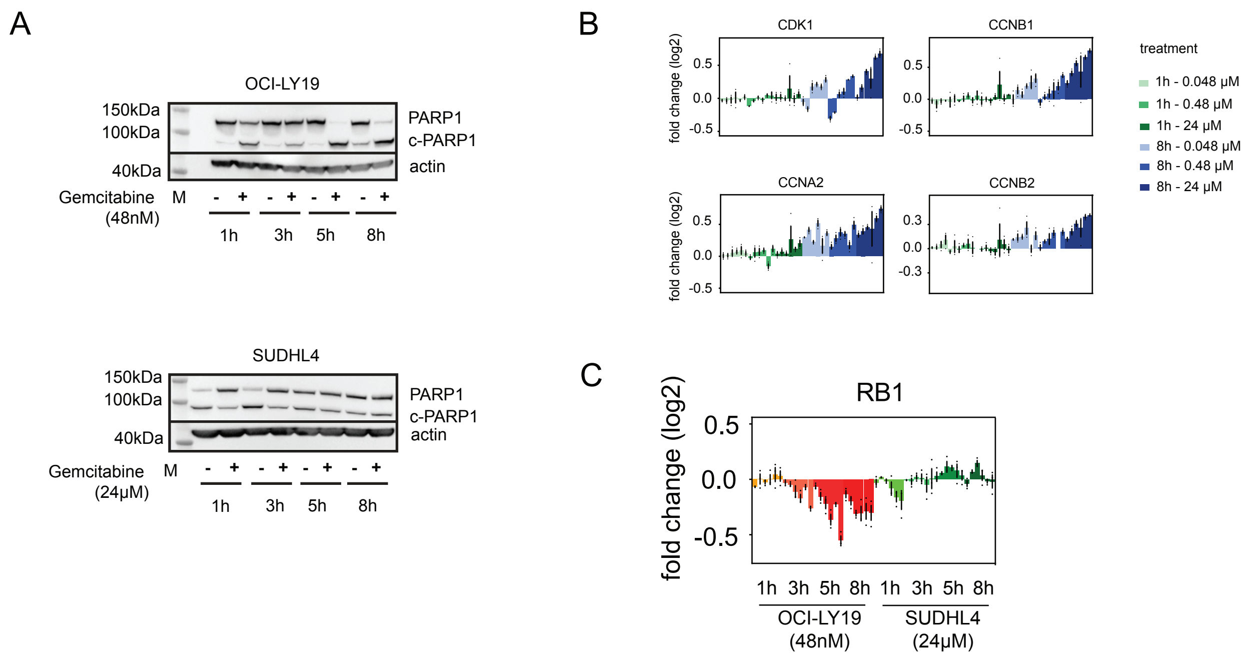

Supplementary Figure 4: Apoptosis in sensitive versus cell cycle progression in resistant cells
(A) Western blot detection of PARP1 and cleaved PARP1 (c-PARP1) levels in OCI-LY19 (left) and SUDHL4 (right) cells after treatment with indicated gemcitabine concentrations for 1h, 3h, 5h, or 8h. Actin was used as loading control. Source data are provided as a Source Data file.(B) IMPRINTS profiles of CDK1, CCNA2, CCNB1 and CCNB2 in SUDHL4 cells treated for 1h (green) or 8h (blue) with 48nM, 480nM and 24μM gemcitabine. Data are presented as mean log2 fold change compared to the reference ±SEM from biological replicates (n=3). Source data are provided as a Source Data file.
(C) IMPRINTS profile of RB1 in gemcitabine sensitive OCI-LY19 (red hues) and resistant SUDHL4 cells (green hues) after 1h, 3h, 5h and 8h of gemcitabine treatment. Data are presented as mean log2 fold change compared to the reference ±SEM from biological replicates (n=3).
Source data are provided as a Source Data file.

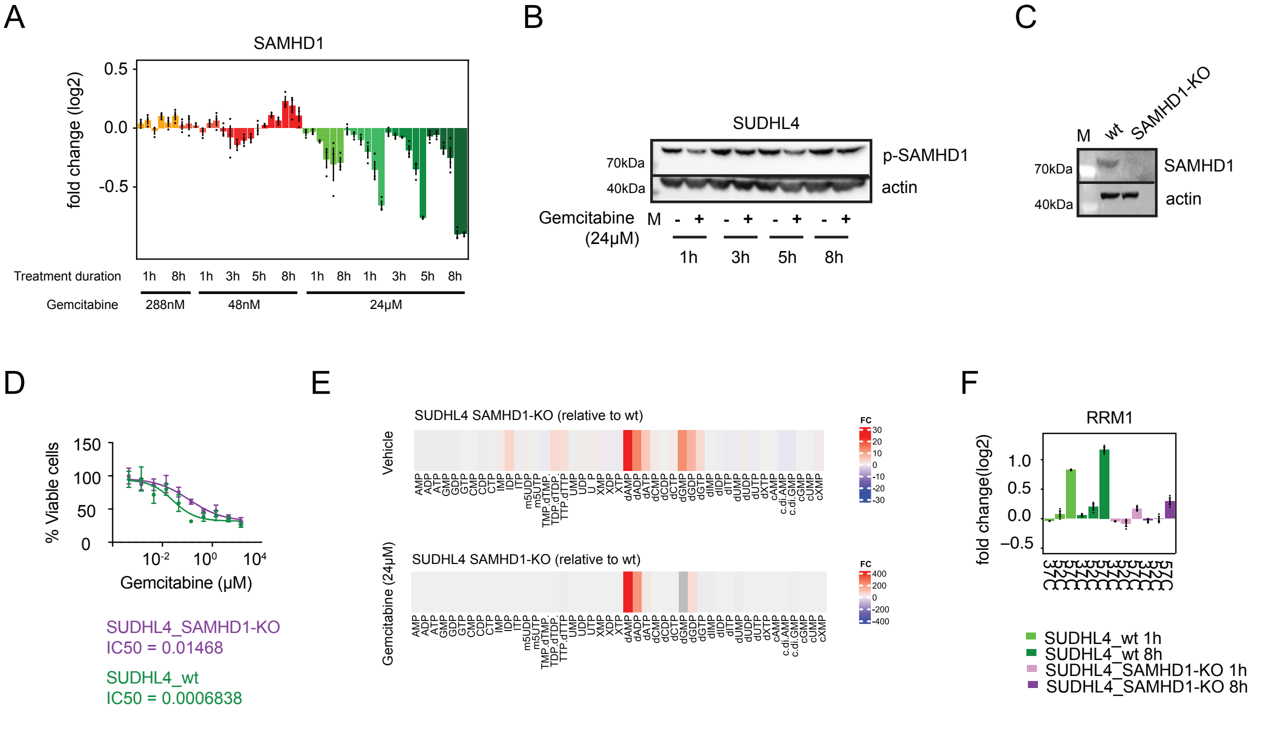

Supplementary Figure 5: Perturbing nucleotide metabolism increases gemcitabine resistance
(A) IMPRINTS profile of SAMHD1 in gemcitabine sensitive OCI-LY19 (red hues) and resistant SUDHL4 cells (green hues) after 1h, 3h, 5h and 8h of gemcitabine treatment. Data are presented as mean log2 fold change compared to the reference ±SEM from biological replicates (n=3). Source data are provided as a Source Data file. (B) Western blot detection of phospho-SAMHD1 in SUDHL4 cells after 1h, 3h, 5h, or 8h of 24µM gemcitabine treatment. Actin was used as loading control. Source data are provided as a Source Data file.(C) Western blot detection of SAMHD1 levels in SUDHL4 SAMHD1 WT and KO cells. Actin was used as loading control. Source data are provided as a Source Data file. (D) Relative viability of SUDHL4 SAMHD1 WT (green) and KO (purple) cells after 72h treatment with increasing concentrations of gemcitabine. Data are presented as mean relative viability compared to the reference ±SEM from biological replicates (n=6). Source data are provided as a Source Data file. (E) LC/MS measurements of deoxyribonucleotides (and ribonucleotides) in SUDHL4 SAMHD1-KO cells treated with vehicle or 24µM gemcitabine for 6h. Graphs show mean relative fold changes as compared to SUDHL4 WT cells from technical replicates (n=3). (F) IMPRINTS profile of RRM1 in SUDHL4 SAMHD1 WT (green) and KO (purple) cells after 1h or 8h gemcitabine treatment. Data are presented as mean log2 fold change compared to the reference ±SEM from biological replicates (n=3).
Source data are provided as a Source Data file.

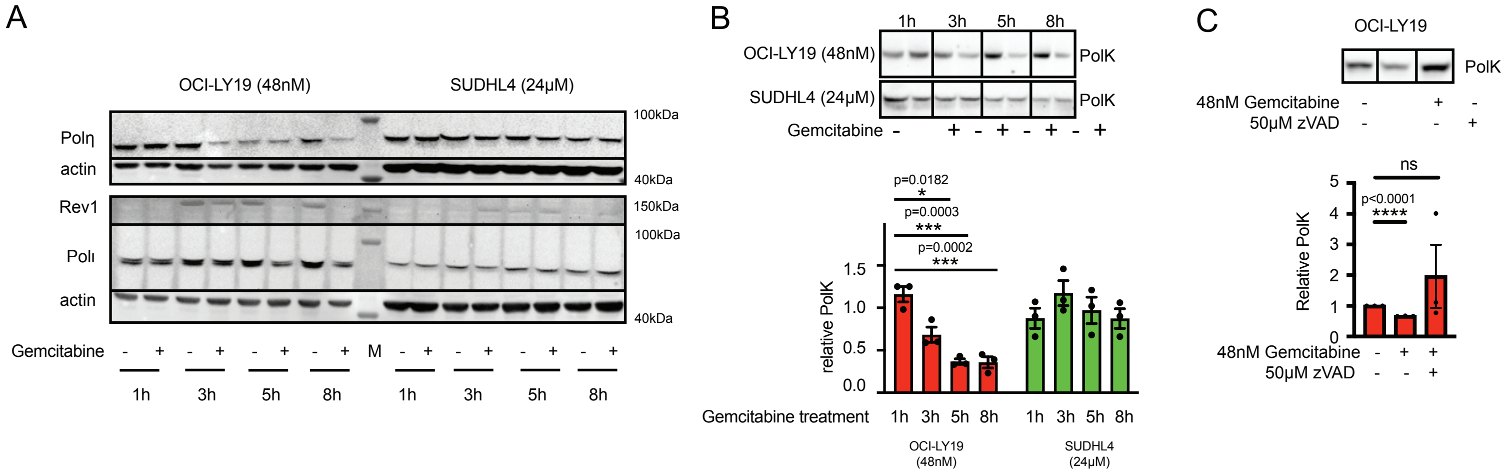

Supplementary Figure 6: Caspase-dependent decrease of Polκ in sensitive cells
(A) Western blot detection of Polη, Rev1 and Polι levels in OCI-LY19 and SUDHL4 cells after treatment with indicated gemcitabine concentrations for 1h, 3h, 5h, or 8h. Actin was used as loading control. Source data are provided as a Source Data file.
(B) Representative western blot (top) and quantification (bottom) of Polκ expression in OCI-LY19 (red) and SUDHL4 (green) cells after 1h, 3h, 5h and 8h of gemcitabine treatment. A two-way ANOVA was performed comparing Gemcitabine treatments at 3h, 5h, and 8h as compared to 1h and data are presented as mean relative fold change compared to the reference ±SEM from biological replicates (n=3). Source data are provided as a Source Data file.
(C) Western blot of Polκ in OCI-LY19 cells after 6h of gemcitabine treatment in the presence or absence of the pan-caspase inhibitor zVAD-FMK. Unpaired, two-tailed t-test comparing vehicle and treatment are shown, respectively. Data presented as mean relative fold change compared to the reference ±SEM from biological replicates (n=3).
Source data are provided as a Source Data file.

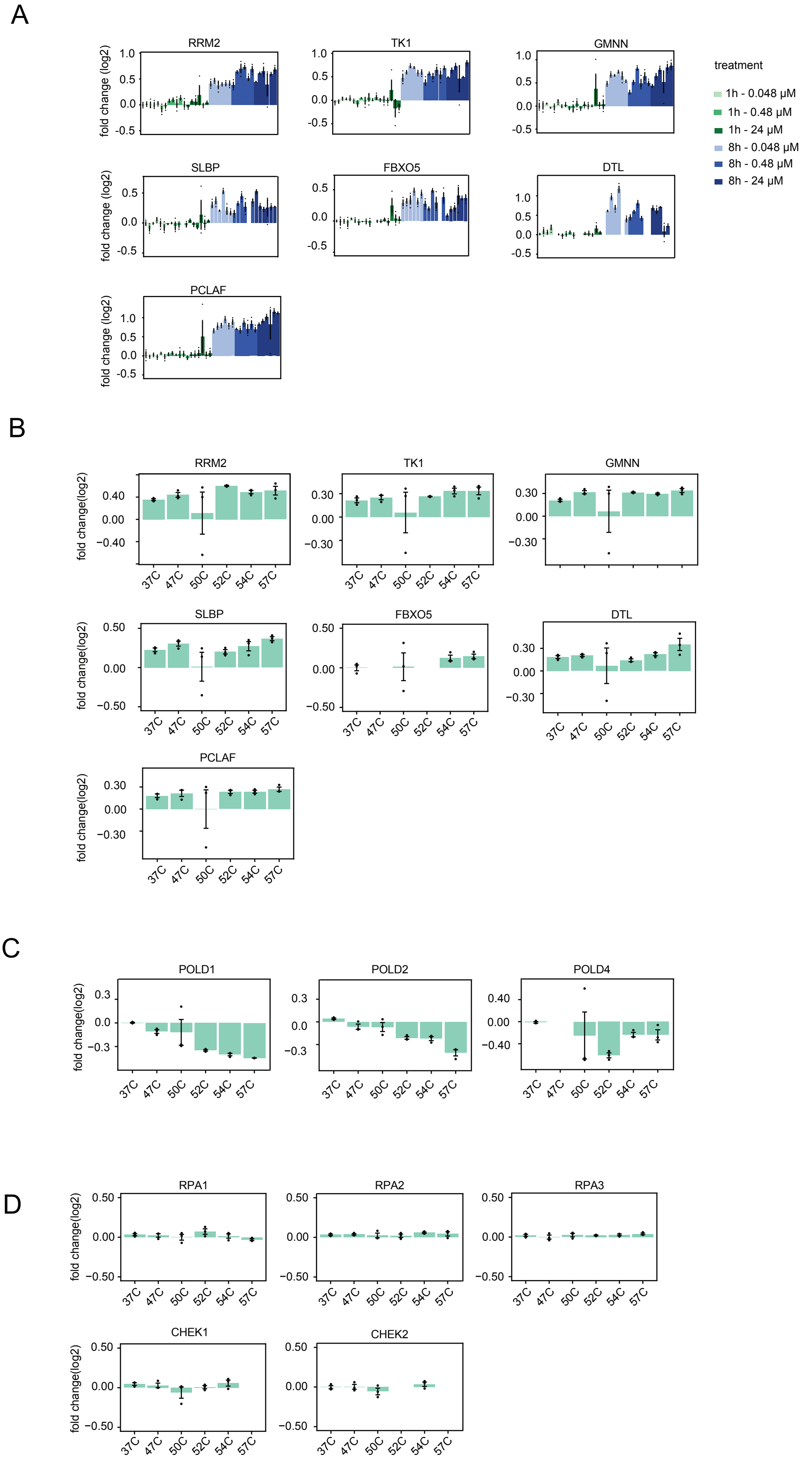

Supplementary Figure 7: IMPRINTS profiles of ADDR and TLS protein ensemble
(A) IMPRINTS profiles of the ADDR proteins in gemcitabine resistance in SUDHL4 cells treated for 1h (green) or 8h (blue) with 48nM, 480nM and 24μM gemcitabine. Data are presented as mean log2 fold change compared to the reference ±SEM from biological replicates (n=3). IMPRINTS profiles of the (B) ADDR protein ensemble, (C) Polδ subunits and (D) proteins involved in stalled replication fork in MDA-MB-231 breast cancer cells treated with 25μM cisplatin for 12h. Data are presented as mean log2 fold change compared to the reference ±SEM from biological replicates (n=3). Source data are provided as a Source Data file.

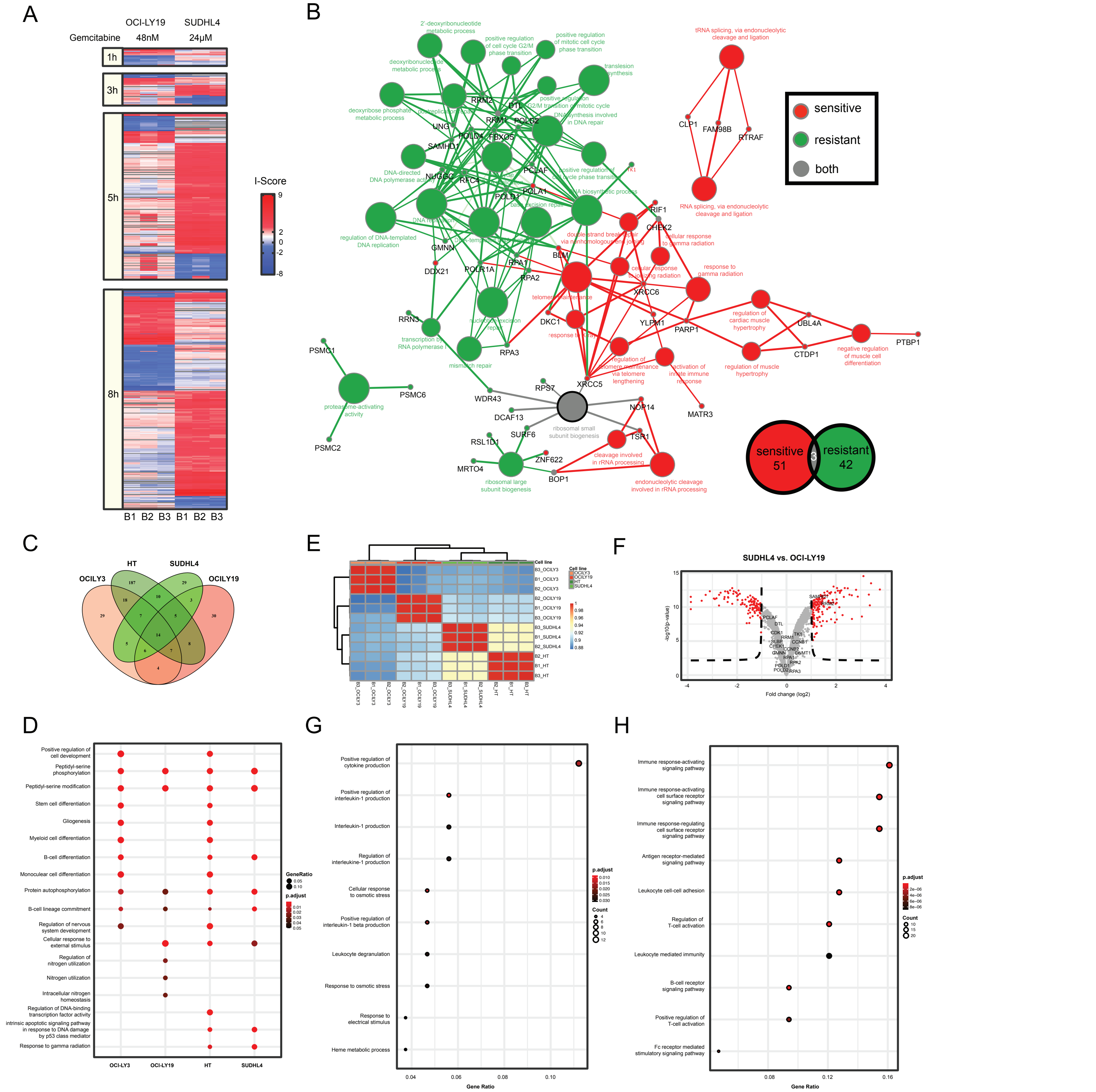

Supplementary Figure 8: Static cell profiling insufficient to identify resistance mechanisms
(A) Heatmap showing the evolution of hit lists at the different time points (1h, 3h, 5h and 8h) after gemcitabine treatment in the sensitive OCI-LY19 cells and resistant SUDHL4 cells. (B) Venn diagram shows overlap of sensitive (red) and resistant (green) hits used for subsequent ClueGO analysis. Only common hits from both sensitive OCI-LY3 and OCI-LY19 (54 hits), and both resistant HT and SUDHL4 (43 hits) cells, after 8h gemcitabine treatment, are used. ClueGO analysis of common hits from sensitive (red) and resistant (green) cells after 8h gemcitabine treatment. Each node represents an enriched GO term whereby the colouring indicates an at least 60% hit contribution from a condition. Hits related to each GO term are depicted in small nodes with black label and coloured accordingly (sensitive = red, resistant = green, both = grey). (C) Venn diagram showing number and overlap of genetic mutations in sensitive (OCI-LY19 and OCI-LY3) and resistant (HT and SUDHL4) cells from the "CCLE Cell Line Gene Mutation Profiles" database. (D) Cell line mutation over-representation analysis of significantly enriched pathways for OCI-LY19, OCI-LY3, HT and SUDHL4 cells. (E) Correlation heat map of 4 cell lines. (F) Volcano plot of quantitative proteomics data in SUDHL4 cells when compared to OCI-LY19. Proteins of interest are labelled with hits depicted in red. Source data are provided as a Source Data file. Cell line quantitative proteomics profiling over-representation analysis of significantly enriched pathways of down- (G) and up-regulated (H) hits in SUDHL4 cells when compared to OCI-LY19.

SUPPLEMENTARY TABLE 1

REAGENT or RESOURCE	SOURCE	IDENTIFIER
**Antibodies**		
Anti-SOD1 rabbit pAb 1:1000	Sigma	HPA001401
Anti-RRM2 1:1000	Santa Cruz Biotechnology	Sc-81850
Anti-TK1 1:1000	GeneTex	GTX113281
anti-GMNN 1:1000	Santa Cruz Biotechnology	Sc-74456
anti-SLBP 1:1000	Invitrogen	PA5-53966
anti-FBXO5 1:1000	Invitrogen	37-6600
anti-DTL 1:1000	Invitrogen	PA5-88380
anti-PCNA 1:1000	Santa Cruz Biotechnology	Sc-56
anti-ubPCNA 1:1000	Cell Signaling Technolgy	D5C7P
Anti-DNMT1 1:1000	Abcam	ab19905
Anti-SAMHD1 1:1000	GeneTex	GTX103751
Anti-phospho-SAMHD1 1:1000	Cell Signaling Technolgy	D7O2M
Anti-pChk1 (S345) 1:1000	Cell Signaling Technology	2348T
Anti-PARP1 1:1000	Santa Cruz Biotechnology	sc-8007
Anti-beta-actin 1:1000	Santa Cruz Biotechnology	Sc-69879
Anti-PolK 1:1000	Santa Cruz Biotechnology	sc-166667
Anti-PolH 1:1000	Santa Cruz Biotechnology	sc-17770
Anti-PolI 1:1000	Santa Cruz Biotechnology	sc-101026
Anti-Rev1 1:1000	Santa Cruz Biotechnology	sc-393022
Anti-rabbit IgG HRP-conjugated secondary antibody 1:5000	Invitrogen	31460
Anti-mouse IgG HRP-conjugated secondary antibody 1:5000	Invitrogen	31430
Anti-goat IgG HRP-conjugated secondary antibody 1:5000	Santa Cruz Biotechnology	Sc-2354
**Chemicals, Peptides, and Recombinant Proteins**		
RPMI-1640 medium	Cytiva	SH30096.01

Heat-inactivated fetal bovine serum (FBS)	Cytiva	SV30160.03
Penicillin-Streptomycin	Gibco	15140-122
MEM Non-Essential Amino Acids	Pan Biotech	P08-32100
L-Glutamine	Gibco	25030-081
Sodium pyruvate	Lonza	13-115E
PBS	Gibco	20012-027
TrypLE Select (1X)	Gibco	12563-029
Gemcitabine	MedChemExpress	HY-B0003
Z-VAD-FMK	Abcam	ab-120487
MG-132	Abcam	ab141003
Cisplatin	MedChemExpress	HY-17394
Cytarabine	MedChemExpress	HY-13605
Cladribine	MedChemExpress	HY-13599
Decitabine	MedChemExpress	HY-A0004
REV7/REV3L-IN-1	MedChemExpress	HY-100468
Halt™ Protease Inhibitor Cocktail, EDTA-Free (100 X)	Thermo Scientific	1861279
Ribonuclease A	Sigma	R6513
NuPAGE™ LDS Sample Buffer (4X)	Thermo Scientific	NP0008
NuPAGE™ Sample Reducing Agent (10X)	Thermo Scientific	NP0009
NuPAGE™ MES SDS Running Buffer (20X)	Thermo Scientific	NP0002
Tris Buffered Saline with 0,05% Tween 20 (TBS-T)	Medicago	09-7510-100
RIPA buffer (Radioimmunoprecipitation assay buffer)	Thermo Scientific	89900

HEPES (hydroxyethyl-piperazineethane-sulphonic acid buffer)	GOLDBIO	H-400-1
Beta-glycerophosphate	Sigma	G9422
Sodium orthovanadate	Sigma	72060
Benzonase Endonuclease	EMD Millipore	1.01695.0001
Triethylammonium bicarbonate buffer (TEAB)	Sigma	T7408
TCEP (tris(2-carboxyethyl)phosphine hydrochloride)	Sinopharm Chemical Reagent Co, Ltd	Xw518054592
2-chloroacetamide (CAA)	Sigma	C0267
Lys-C	Wako Chemicals Ltd	129-02541
Trypsin	Promega	V5117
TFA (Trifluoroacetic acid)	Sigma	T6508
Tris	1st Base	BIO-1400
Acetonitrile	Sigma	T7408
TMT10PLEX Isobaric Label Reagent Set	Thermo Scientific	90110
Ammonia solution 25%	Merck	5.33003.0050
Propidium Iodide	Sigma	P4170
0.1% Formic Acid in ACETONITRILE	FISHER US	ZZLFS/LS120-212
0.1% Formic Acid in Water	FISHER US	ZZLFS/HB523-4
LC-MS hypergrade acetonitrile (ACN)	Merck	100029
LC-MS grade acetic acid	Merck	533001
**Critical Commercial Assays**		
BCA assay	Thermo Scientific	23227
Clarity Western ECL substrate	Bio-Rad	170-5060

MTT	Cayman Chemical	21795-1
**Deposited Data**		
MS-CETSA raw data	ProteomeXchange via the jPOST repository	http://proteomecentral.proteomexchange.org/ Dataset identifiers PXD054912, PXD054911, PXD054910, PXD054909, PXD054908, PXD054907, PXD054903, PXD054902, PXD054901, PXD054854, PXD054853, PXD054852, PXD055016, PXD055015
Experimental Models: Cell Lines		
SUDH-L4	ATCC	CRL-2957
HT	ATCC	CRL-2260
OCI-LY3	ATCC	
OCI-LY19	ATCC	
MDA-MB-231	ATCC	CRM-HTB-26
**Software and Algorithms**		
ImageLab^{TM} software	BioRad	https://www.bio-rad.com/

Xcalibur v.4.0	Thermo Scientific	https://www.thermofisher.com/us/en/home.html
Proteome Discoverer v.2.5	Thermo Scientific	https://www.thermofisher.com/us/en/home.html
MASCOT 2.6.0	MATRIX SCIENCE	http://matrixscience.com
Sequest HT	Thermo Scientific	https://www.thermofisher.com/us/en/home.html
RStudio v.1.2.5033	RStudio	https://www.rstudio.com
R v.3.6.3	The R Foundation	https://www.r-project.org/
mineCETSA 0.3.8.7	Lim et al., 2018 *(39)*	https://github.com/nkdailingyun/mineCETSA
IMPRINTS.CETSA 1.0.4	Gerault et al., 2024 *(38)*	https://github.com/nkdailingyun/IMPRINTS.CETSA
IMPRINTS.CETSA.app 3.4.2	Gerault et al., 2024 *(38)*	https://github.com/mgerault/IMPRINTS.CETSA.app
Cytoscape 3.9.2	Cytoscape	http://cytoscape.org
ClueGO plugin v2.5.1 for Cytoscape	Bindea et al., 2009	http://apps.cytoscape.org/apps/cluego
GraphPad Prism v.8.3.0	GraphPad Software	https://www.graphpad.com/
Biorender		www.biorender.com

FlowJo v.9.3.2	FLOWJO, LLC	https://www.flowjo.com
I-Control 1.9	Tecan	https://lifesciences.tecan.com/plate_readers/infinite_200_pro?p=tab--3
**Other**		
MicroAmp™ Fast 96-well Reaction plate	Applied Biosystems	4346907
Falcon® 150cm² cell culture flask	Corning	355001
Falcon® 75cm² cell culture flask	Corning	353136
Falcon® 25cm² cell culture flask	Corning	353109
96-well plates (black)	Greiner	655090
NuPAGE 4–12% Bis-Tris midi gel	Invitrogen	WG1403BX10
iBlot 2 NC Regular Stacks	Invitrogen	IB23001
Non-fat milk powder	Semper AB	N/A
Oasis HLB 1cc (10mg) extraction cartridges	Waters	186000383
Xbridge Peptide BEH C18, 300 Å, 3.5 µm, 2.1 mm × 250 mm column	Waters	186003610
Zorbax 300 Extend C-18 4.6 mm × 250 mm column	Agilent	770995-902
50 cm x 75 µm(ID) EASY-Spray analytical column	Thermo Scientific	ES903
Veriti™ 96-Well Thermal Cycler	Applied Biosystems	P/N 4375786
XCell4 SureLock™ Midi-Cell	Invitrogen	Cat#WR0100
iBlot 2 system	Invitrogen	Cat#IB21001

ChemiDoc^{TM} XRS+ imaging system	BioRad	Universal Hood III
SpeedVac vacuum concentrator	Thermo Scientific	P/N SPD111V-230, 61010-1, and RV5 A65313906
Dionex UltiMate 3000 UPLC system	Thermo Scientific	P/N 5041.0010, 5826.0020, and 5035.9245
Q Exactive mass spectrometer	Thermo Scientific	IQLAAEGAAPFA LGMBFZ
Veriti 96 well Thermal cycler	Invitrogen	4375786
ChemiDoc MP Imaging System	Bio-Rad	1708280
50cmx75µM (ID) EASY-Spray analytical column	ThermoFisher Scientific	P/N ES803
High pH reverse phase Zorbax 300 Extend C-18 4.6mm x 250mm column	Agilent	P/N 770995-902
Liquid chromatography AKTA microsystem	GE Healthcare	28948303
Oasis HLB 96-well plate	Waters	WAT058951
Tecan Infinite M200 Plate Reader	Tecan	30016056
Eppendorf concentrator plus	Eppendorf AG Hamburg	5305000304
Centrifuge 5424 R	Eppendorf	5404000014
