# Supplementary material for: MS CETSA deep functional proteomics uncovers DNA repair programs leading to gemcitabine resistance
Source: Nat Commun. 2025 May 7;16:4234. doi: 10.1038/s41467-025-59505-8 (PMC12059070; doi:10.1038/s41467-025-59505-8)
Supplement: Supplementary file 2 — Description of Additional Supplementary Files [file 41467_2025_59505_MOESM2_ESM.pdf]

Description of Additional Supplementary Files

File name: Supplementary Data 1

Description: Summary of MS-CETSA datasets generated in this study, including number of identified proteins and hits for respective conditions

File name: Supplementary Data 2

Description: MOLM-16 Protein Turnover Rates (half-life in hours) and annotation as short-lived (<12h), fast (12h – 24h), moderate (24h – 72h), or slow (>72h).
