# Supplementary material for: MS CETSA deep functional proteomics uncovers DNA repair programs leading to gemcitabine resistance
Source: Nat Commun. 2025 May 7;16:4234. doi: 10.1038/s41467-025-59505-8 (PMC12059070; doi:10.1038/s41467-025-59505-8)
Supplement: Supplementary file 5 — Reporting Summary [file 41467_2025_59505_MOESM5_ESM.pdf]

nature portfolio

Corresponding author(s): Prof. Pär Nordlund, Dr. Nayana Prabhu, Dr. Wai Leong Tam

Last updated by author(s): Mar 11, 2025

Reporting Summary

Nature Portfolio wishes to improve the reproducibility of the work that we publish. This form provides structure for consistency and transparency in reporting. For further information on Nature Portfolio policies, see our Editorial Policies and the Editorial Policy Checklist.

Statistics

For all statistical analyses, confirm that the following items are present in the figure legend, table legend, main text, or Methods section.

n/a	Confirmed	
☐	☒	The exact sample size (n) for each experimental group/condition, given as a discrete number and unit of measurement
☐	☒	A statement on whether measurements were taken from distinct samples or whether the same sample was measured repeatedly
☐	☒	The statistical test(s) used AND whether they are one- or two-sided<br>*Only common tests should be described solely by name; describe more complex techniques in the Methods section.*
☒	☐	A description of all covariates tested
☒	☐	A description of any assumptions or corrections, such as tests of normality and adjustment for multiple comparisons
☐	☒	A full description of the statistical parameters including central tendency (e.g. means) or other basic estimates (e.g. regression coefficient) AND variation (e.g. standard deviation) or associated estimates of uncertainty (e.g. confidence intervals)
☐	☒	For null hypothesis testing, the test statistic (e.g. F, t, r) with confidence intervals, effect sizes, degrees of freedom and P value noted<br>*Give P values as exact values whenever suitable.*
☒	☐	For Bayesian analysis, information on the choice of priors and Markov chain Monte Carlo settings
☐	☒	For hierarchical and complex designs, identification of the appropriate level for tests and full reporting of outcomes
☒	☐	Estimates of effect sizes (e.g. Cohen's d, Pearson's r), indicating how they were calculated

Our web collection on statistics for biologists contains articles on many of the points above.

Software and code

Policy information about availability of computer code

Data collection	Cell viability assays were measured using I-Control 1.9 software for Tecan InfiniteM200 plate reader. Flow cytometry data was collected using BD FACSDiva software. Mass spectrometry data collection was performed using commercially available software developed by the instrument manufacturer as follows: Chromeleon™ software was used for operating the Dionex UltiMate 3000 UPLC system used in the fractionation step prior to MS. Xcalibur™ software was used for MS data aquisition on a Q Exactive mass spectrometer (Thermo Scientific) . Proteome Discoverer 2.5 software (Thermo Scientific), using Sequest HT (Thermo Scientific) was utilized for protein identification and quantification. For western blot experiments, data collection and quantification was performed using the ImageLab™ software available from BioRad.
Data analysis	For flow cytometric analysis raw data was imported into FlowJo v10.8 to perform gating strategies. Drug synergy scoring of combination drug assay was calculated with SynergyFinder package v3.10.3 using Zero Interaction Potency (ZIP) as the reference model. For the MS-CETSA experiments, the quantified protein abundances were imported into the R environment (http://www.R-project.org/) to facilitate the data analysis and visualisation. Data cleaning, normalisation, and calculations of protein abundance and thermal stability differences in each condition were performed using IMPRINTS.CETSA and IMPRINTS.CETSA.app packages, which are available online at https://github.com/nkdailingyun/IMPRINTS.CETSA and https://github.com/mgerault/IMPRINTS.CETSA.app. These package include in-depth instructions for use and descriptions of the functions contained in the package and the arguments for each function. Protein-protein interaction networks for hits were obtained by importing a list of Uniprot IDs into Cytoscape (http://cytoscape.org). Comparative GO analysis was performed using the ClueGO plug-in in Cytoscape (http://apps.cytoscape.org/apps/cluego). All graphs were generated using GraphPad Prism (Version 10.1.1), R environment (Rv4.2.2, RStudio 2022.02.0 Build 443), Cytoscape (v.3.9.1), ClueGO v2.5.1, or Biorender (Fig 1B, Fig2, Fig 5F, Fig7)

For manuscripts utilizing custom algorithms or software that are central to the research but not yet described in published literature, software must be made available to editors and reviewers. We strongly encourage code deposition in a community repository (e.g. GitHub). See the Nature Portfolio guidelines for submitting code & software for further information.

Data

Policy information about [availability of data](#)

All manuscripts must include a [data availability statement](#). This statement should provide the following information, where applicable:

- Accession codes, unique identifiers, or web links for publicly available datasets
- A description of any restrictions on data availability
- For clinical datasets or third party data, please ensure that the statement adheres to our [policy](#)

All data generated or analysed during this study are included in this published article (and its supplementary information files).

Research involving human participants, their data, or biological material

Policy information about studies with [human participants or human data](#). See also policy information about [sex, gender (identity/presentation), and sexual orientation](#) and [race, ethnicity and racism](#).

Reporting on sex and gender	As this study includes two biopsies of clinical DLBCL patients for ex vivo drug treatment, sex and gender of the patients were not relevant and thus not taken into consideration when samples were collected.
Reporting on race, ethnicity, or other socially relevant groupings	N.A.
Population characteristics	N.A.
Recruitment	This study includes two biopsies of clinical DLBCL patients who have relapsed after first line therapy and have not been previously treated with gemcitabine.
Ethics oversight	A*STAR IRB: 2021-140

Note that full information on the approval of the study protocol must also be provided in the manuscript.

Field-specific reporting

Please select the one below that is the best fit for your research. If you are not sure, read the appropriate sections before making your selection.

☒ Life sciences ☐ Behavioural & social sciences ☐ Ecological, evolutionary & environmental sciences

For a reference copy of the document with all sections, see [nature.com/documents/nr-reporting-summary-flat.pdf](#)

Life sciences study design

All studies must disclose on these points even when the disclosure is negative.

Sample size	No sample size calculations were performed for the experiments included in this paper. The data included in the paper represent three biological experimental repeats (n=3) with the exception of the ITDR CETSA experiments (Fig 1E and Fig 6) where two technical replicates were included (n=2) and the flow cytometric analysis with four biological replicates (n=4). We determined that biological replicates would be appropriate for our experiments (when possible) as this would reflect sufficiently on the reproducibility of the results and allow the application of statistical analysis where appropriate.
Data exclusions	No data were excluded.
Replication	All attempts at replicating the data were successful and therefore included in the paper.
Randomization	The nature of our study did not require any randomization to be performed.
Blinding	The nature of our study did not require any considerations regarding blinding.

Reporting for specific materials, systems and methods

We require information from authors about some types of materials, experimental systems and methods used in many studies. Here, indicate whether each material, system or method listed is relevant to your study. If you are not sure if a list item applies to your research, read the appropriate section before selecting a response.

Materials & experimental systems

n/a	Involved in the study
☐	☒ Antibodies
☐	☒ Eukaryotic cell lines
☒	☐ Palaeontology and archaeology
☒	☐ Animals and other organisms
☒	☐ Clinical data
☒	☐ Dual use research of concern
☒	☐ Plants

Methods

n/a	Involved in the study
☒	☐ ChIP-seq
☐	☒ Flow cytometry
☒	☐ MRI-based neuroimaging

Antibodies

Antibodies used	For protein detection with western blot, the following antibodies were used: Santa Cruz Biotechnology: anti-RRM2 (sc-81850), anti-GMNN (sc-74456), anti-PCNA (sc-56), anti-PARP1 (sc-8007), anti-PolK (sc-166667), anti-PolH (sc-17770), anti-PolI (sc-101026), anti-Rev1 (sc-393022), anti-beta actin (sc-69879), anti-goat IgG HRP-conjugated secondary antibody (sc-2354). Invitrogen: anti-SLBP (PA5-53966), anti-FBXO5 (37-6600), anti-DTL (PA5-88380), anti-rabbit IgG HRP-conjugated secondary antibody (31460), anti-mouse IgG HRP-conjugated secondary antibody (31430). Cell Signaling Technology: anti-phospho SAMHD1 (D7O2M), anti-phospho Chek1 (S345) (2348T), anti-ubPCNA (D5C7P). Genetex: anti-TK1 (GTX113281), anti-SAMHD1 (GTX103751). Abcam: anti-DNMT1 (ab19905). Sigma: anti-SOD1 (HPA001401).
Validation	Antibodies were validated by the manufacturer for applications such as WB, IF or IHC in multiple cell lines and/or different types of human tissues. The manufacturer's website also lists peer-reviewed publications where antibodies have been referred to.

Eukaryotic cell lines

Policy information about cell lines and Sex and Gender in Research

Cell line source(s)	Several cancer cell lines of human origin were purchased from ATCC: SUDHL4 (CRL-2957), HT (CRL-2260), MDA-MB-231 (CRM-HTB-26). Cell lines OCI-LY19 and OCI-LY3 were obtained from the lab 661 of Manikandan Lakshmanan at IMCB, Singapore.
Authentication	For all cell lines, the morphology of the cells was frequently monitored under the microscope to ensure that the culture has its correct and unusual appearance.
Mycoplasma contamination	Utilized cell lines for this study have tested negative for mycoplasma contamination.
Commonly misidentified lines (See ICLAC register)	Our study did not include any cell lines that are commonly misidentified.

Plants

Seed stocks	N.A.
Novel plant genotypes	N.A.
Authentication	N.A.

Flow Cytometry

Plots

Confirm that:

☐ The axis labels state the marker and fluorochrome used (e.g. CD4-FITC).

☐ The axis scales are clearly visible. Include numbers along axes only for bottom left plot of group (a 'group' is an analysis of identical markers).

☐ All plots are contour plots with outliers or pseudocolor plots.

☐ A numerical value for number of cells or percentage (with statistics) is provided.

Methodology

Sample preparation	Cell lines were seeded at 0.5x10^6 cells/ml of media and preconditioned in complete RPMI with 2% FBS for 24h. The cells were then treated with either vehicle or drug at their respective final concentrations and incubated at 37°C and 5% CO_2 for indicated time points. Cells were pelleted for 4min at 400xg, washed with PBS. Cells were fixed in 70% ethanol overnight, washed twice with cold PBS, then resuspended in PI staining solution (100µg/ml ribonuclease A, 50µg/ml PI in PBS) and incubated in the dark for at least 30min at room temperature, followed by flow cytometric analysis.
Instrument	LSR II (BD Biosciences, UK)
Software	Data collection was performed using BD FacsDIVA Software, subsequent data analysis was done using FlowJo (FlowJo, LLC, USA)
Cell population abundance	*Describe the abundance of the relevant cell populations within post-sort fractions, providing details on the purity of the samples and how it was determined.*
Gating strategy	Gating strategy is not provided in the supplementary information, but can be submitted in the final version for publication if requested.

☐ Tick this box to confirm that a figure exemplifying the gating strategy is provided in the Supplementary Information.
